# Polyploid tubular cells initiate a TGF-β1 controlled loop that sustains polyploidization and fibrosis after acute kidney injury

**DOI:** 10.1152/ajpcell.00081.2023

**Published:** 2023-08-29

**Authors:** Letizia De Chiara, Roberto Semeraro, Benedetta Mazzinghi, Samuela Landini, Alice Molli, Giulia Antonelli, Maria Lucia Angelotti, Maria Elena Melica, Laura Maggi, Carolina Conte, Anna Julie Peired, Luigi Cirillo, Valentina Raglianti, Alberto Magi, Francesco Annunziato, Paola Romagnani, Elena Lazzeri

**Affiliations:** ^1^Department of Experimental and Clinical Biomedical Sciences “Mario Serio”, University of Florence, Florence, Italy; ^2^Department of Experimental and Clinical Medicine, University of Florence, Florence, Italy; ^3^Nephrology and Dialysis Unit, Meyer Children’s University Hospital, IRCCS, Florence, Italy; ^4^Medical Genetics Unit, Meyer Children’s University Hospital, IRCCS, Florence, Italy; ^5^Department of Information Engineering, University of Florence, Florence, Italy; ^6^Flow Cytometry Diagnostic Center and Immunotherapy (CDCI), Careggi University Hospital, Florence, Italy

**Keywords:** CKD, fibrosis, polyploidy, TGF-β1, tubular epithelial cells

## Abstract

Polyploidization of tubular cells (TC) is triggered by acute kidney injury (AKI) to allow survival in the early phase after AKI, but in the long run promotes fibrosis and AKI-chronic kidney disease (CKD) transition. The molecular mechanism governing the link between polyploid TC and kidney fibrosis remains to be clarified. In this study, we demonstrate that immediately after AKI, expression of cell cycle markers mostly identifies a population of DNA-damaged polyploid TC. Using transgenic mouse models and single-cell RNA sequencing we show that, unlike diploid TC, polyploid TC accumulate DNA damage and survive, eventually resting in the G1 phase of the cell cycle. In vivo and in vitro single-cell RNA sequencing along with sorting of polyploid TC shows that these cells acquire a profibrotic phenotype culminating in transforming growth factor (TGF)-β1 expression and that TGF-β1 directly promotes polyploidization. This demonstrates that TC polyploidization is a self-sustained mechanism. Interactome analysis by single-cell RNA sequencing revealed that TGF-β1 signaling fosters a reciprocal activation loop among polyploid TC, macrophages, and fibroblasts to sustain kidney fibrosis and promote CKD progression. Collectively, this study contributes to the ongoing revision of the paradigm of kidney tubule response to AKI, supporting the existence of a tubulointerstitial cross talk mediated by TGF-β1 signaling produced by polyploid TC following DNA damage.

**NEW & NOTEWORTHY** Polyploidization in tubular epithelial cells has been neglected until recently. Here, we showed that polyploidization is a self-sustained mechanism that plays an important role during chronic kidney disease development, proving the existence of a cross talk between infiltrating cells and polyploid tubular cells. This study contributes to the ongoing revision of kidney adaptation to injury, posing polyploid tubular cells at the center of the process.

## Graphical abstract

**Figure:**
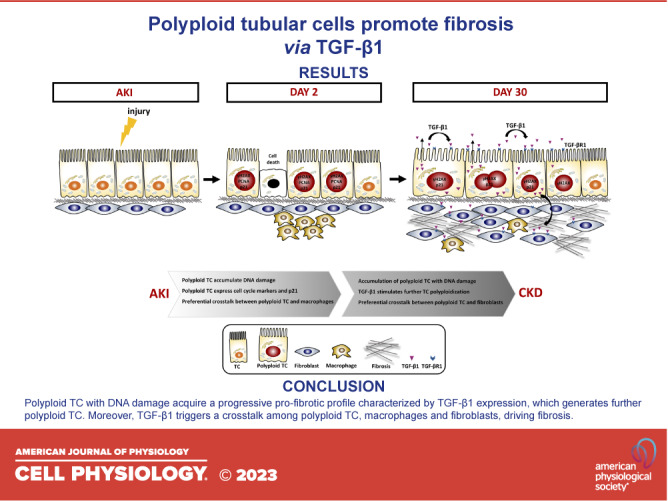

## INTRODUCTION

Acute kidney injury (AKI) is characterized by a sudden kidney failure accompanied by a transient and persistent decrease of kidney functionality (1). It is regarded as an important risk factor for chronic kidney disease (CKD) development. Renal fibrosis, especially tubulointerstitial fibrosis, is the final manifestation of CKD (2, 3) and is characterized by an excessive synthesis and deposition of extracellular matrix (ECM) associated with inflammatory infiltration, tubular epithelial cells (tubular cells, TC) damage, fibroblast activation, and microvasculature rarefaction (3). Although no targeted therapy yet exists to slow the progression of tubulointerstitial fibrosis (3), recent findings contributed to clarifying the cellular and molecular mechanisms underlying its development and progression, posing TC at the center of this process (4–6). Accordingly, we have demonstrated that fibrosis and senescence are trade-offs of TC polyploidy occurring immediately after AKI to support fast kidney function recovery, but promoting consequent CKD (4). However, the mechanisms turning TC polyploidy to senescence and fibrosis still need to be elucidated. Among the many pathways controlling fibrosis deposition, a prominent role is played by the transforming growth factor-β1 (TGF-β1) (7). TGF-β1 is a pleiotropic cytokine that is involved in regulating a broad range of cellular processes (7, 8). In the liver, TGF-β1 is a major inducer of hepatocyte polyploidization (9) and polyploid megakaryocytes are the primary source of TGF-β1 in patients with primary myelofibrosis (10), suggesting the existence of a regulatory loop between fibrosis and polyploidization (10). In addition, DNA damage and genome instability were shown to trigger TGF-β1 production initiating a vicious circle that leads to fibroblast activation and fibrosis development in the intestine (11).

Here, we aimed to investigate the role of polyploid TC in promoting tubulointerstitial fibrosis and CKD. By using single-cell RNA-sequencing (scRNA-Seq) analysis in vitro and in vivo, as well as transgenic mice and in vitro culture, we observed that accumulation of DNA damage in polyploid TC progressively increases after injury, culminating in the acquisition of a profibrotic profile and of TGF-β1 expression that maintains TC polyploidization via YAP1. Moreover, an interactome analysis along with in vitro experiments revealed the existence of a progressive cross talk between polyploid TC, macrophages, and fibroblasts, mediated by TGF-β1 signaling. Collectively, these findings contribute to the ongoing revision of the paradigm of kidney tubule response to AKI, supporting the existence of a tubulointerstitial cross talk mediated by TGF-β1 signaling produced by polyploid TC following DNA damage.

## MATERIALS AND METHODS

### Mice

To visualize the cell cycle progression of Pax8+ TC, the Pax8.rtTA;TetO.Cre;R26.FUCCI2aR (Pax8/FUCCI2aR) mouse model was used. This model was obtained by crossing Pax8.rtTA;TetO.Cre mice (4, 12) with mice harboring the Fluorescent Ubiquitin-based Cell cycle Indicator (FUCCI2aR) Cre-dependent reporter [European Mouse Mutant Archive (EMMA), INFRAFRONTIER-I3, Neuherberg-München, Germany], which consists of a bicistronic Cre-activable reporter of two fluorescent proteins whose expression alternates based on cell cycle phase: mCherry-hCdt1 (30/120) (red), expressed by nuclei of cells in G1 phase, and mVenus-hGem (1/110) (green), expressed by nuclei of cells in S/G2/M. Cells can also appear as yellow at the G1/S boundary (13). Mice were developed on a full C57Bl/6 background (4, 12). Reporter expression was induced in male mice at 5 wk of age with doxycycline treatment for 10 days followed by 1 wk of washout as previously described (4, 12). After that, mice underwent a unilateral ischemia reperfusion injury (uni-IRI) for 30 min, and were then euthanized at *days 2* and *30* after uni-IRI. Sham-operated mice were used as controls. Animal experiments were approved by the Institutional Review Board and by the Italian Ministry of Health and performed in accordance with institutional, regional, and state guidelines and in adherence to the National Institutes of Health Guide for the Care and Use of Laboratory Animals. Mice were housed in a specific pathogen-free facility with free access to chow and water and a 12-h day/night cycle. The references to the ethics approvals are the following: 689/2019-PR and 864/2021-PR.

### Genotyping

Genotyping was performed as previously shown (4, 12). In brief, tail biopsies were incubated overnight at 55°C in lysis reagent, centrifuged, and DNA extracted using isopropanol (Merck). Primers and PCR parameters were obtained from Jackson Laboratory online resources of the relative strain purchased or from previously reported experimental procedures (4).

### Unilateral Ischemia-Reperfusion Injury

Renal ischemia was performed on male mice as previously described (4, 12, 14). Briefly, mice were anesthetized and the left kidney was then externalized and the renal artery was clamped for 30 min. After clamp removal, the muscle layer was sutured, followed by the closure of the skin wound with metal clips. Sham-operated mice underwent the same surgical procedure without left renal artery clamping.

### Blood Urea Nitrogen Quantification

Kidney function was assessed at different time points by collecting a small amount of blood from mice with a metal lancet from the submandibular plexus to measure blood urea nitrogen (BUN) levels. Blood parameters were measured in EDTA anticoagulated plasma samples using Reflotron (Roche Diagnostics), according to the manufacturer’s protocols.

### FACS Analysis on Mouse Kidney

Cell cycle analysis was performed on total FUCCI2aR cells (mCherry+ and mVenus+ cells). Kidneys were processed to obtain a single-cell suspension as previously reported (4, 12). Briefly, kidneys were minced and the digested kidneys were centrifuged, digested, and stained as previously shown (4, 12). Cells were then incubated with 4′,6-diamidino-2-phenylindole (DAPI, 1:2,000, Thermo Fisher Scientific), to perform the DNA content analysis. The assessment of polyploid TC was performed using a MacsQuant instrument (Miltenyi Biotec). Anti-DsRed pAb (Clontec, 632496) was used to mark mCherry+ TC followed by incubation with Alexa Fluor 647-secondary antibody; mVenus+ TC were identified following incubation with anti GFP-AlexaFluor 488 pAb (Thermo Fisher Scientific, A21311). Polyploid TC were defined as mCherry+ or mCherry+mVenus+ cells with a DNA content ≥4C and mVenus+ with a DNA content ≥8C. To detect mVenus+ TC with DNA damage, H2A histone family member X (γH2AX, Thermo Fisher Scientific, 14-9865-82) was incubated for 1 h followed by anti mouse IgG1-Alexa Fluor 647 secondary antibody. To detect mCherry+ TC with DNA damage, γH2AX was incubated for 1 h followed by anti mouse IgG1-Alexa Fluor 488 secondary antibody. Cells were then incubated with DAPI to perform the DNA content analysis. The percentage of polyploid and diploid TC with DNA damage was calculated on the total % of mVenus+ or mCherry+ cells. Diploid versus polyploid fraction was determined based on DNA content. Specifically, mCherry+ TC with 2C DNA content or mVenus+ TC with up to 4C DNA content were considered diploid cells. mCherry+ TC with ≥4C DNA content or mVenus+ TC with ≥8C DNA content were considered polyploid cells. Alexa Fluor 647-secondary antibody was excited by a 633 nm laser line, GFP was excited by a 488 nm laser line, and DAPI was excited by a laser at 405 nm. Gating strategy to exclude cell doublets was performed as previously published (4). All isotype controls are shown in Supplemental Figs. S1, S3, and S5 (all Supplemental material is available at https://doi.org/10.6084/m9.figshare.22200187). Data were analyzed by FlowLogic software (FlowLogic 7.2.1, Inivai Technology).

### hPTC Culture, Virus Transduction, Fluorescence-Activated Cell Sorting, TGF-β1, Verteporfin, and Fresolimumab Treatments

Human proximal tubular cells (hPTC) (ATCC-PCS-400-010) were maintained in renal epithelial growth medium (REGM) (Lonza, CC-3190). hPTC were seeded at a density of 10^5^ cells/6-well. The following day, cells were transduced with a pRetroX-G1-Red (Clontech, 631436) to allow the identification of cells in the G1 phase. A multiplicity of infection (MOI) of 10 was used (Retro-X qRT-PCR Titration Kit, 631453) according to the manufacturer’s instructions. In this plasmid, the cell cycle indicator hCdt1 (30–120) is tagged with the red fluorescent protein mCherry. After transduction, cells are referred to as hPTC-mCherry. These cells were trypsinized (Euroclone) at *passage 2* after transduction, fixed, and stained for FACS as previously described (4). To detect mCherry+ hPTC, cells were incubated with anti-DsRed (1:25, Clontech, 632496) or isotype control and then incubated with Alexa Fluor 647 goat anti-rabbit (1:100, Thermo Fisher Scientific, A-21245). hPTC were then incubated with DAPI (1:2,000, Thermo Fisher Scientific) to perform the DNA content analysis and analyzed on a MacsQuant instrument (Miltenyi Biotec). In the verteporfin experiment, Alexa Fluor 488-goat anti rabbit (1:100, Thermo Fisher Scientific, A21311) was used. Alexa Fluor 488-secondary antibody was excited by a 488 nm laser line, Alexa Fluor 647 secondary antibody was excited by a 635 nm laser line, and DAPI was excited by a 405 nm laser line. Cell cycle analysis and gating strategy to exclude cell doublets were performed on total hPTC as previously published (4). Polyploid hPTC were defined as mCherry+ cells with a DNA content ≥4C. In the sorting experiment, the same protocol was applied but all the antibodies were diluted in 0.5% saponin (Merck) with the addition of 1:100 RNAase inhibitor (Applied Biosystems, N8080119). The solutions were prepared in RNAase-free PBS and the procedure was carried out on ice. Following DAPI incubation, hPTC were sorted on the FACSAria III BD (Bioscience). Alexa Fluor 647-secondary antibody was excited by a 633 nm laser line, DAPI was excited by a 405 nm laser line. Data were analyzed by FacsDiva software (Beckman Coulter). In additional experiments, cells were seeded and the day after treated with TGF-β1 (Peprotech) at a concentration of 10 ng/mL or vehicle (10 nM citric acid). Fresolimumab (HY-P99020, DBA dissolved in DMSO) was incubated at the concentration of 10 µg/mL (15) for 1 h before TGF-β1 stimulation. DMSO (Merck) was used as vehicle control. Effective block of TGF-β1 pathway activation was tested by incubating hPTC with Fresolimumab or vehicle control for 48 h. Cells were then harvested followed by RNA extraction and real-time analysis of relevant targets was performed. For verteporfin treatment (Biotechne, 5305), cells were stimulated with a verteporfin concentration of 0.6 µM 1 h prior to TGF-β1 treatment. Cells were then harvested after 48 h. DMSO (Merck) was used as vehicle control. After 48 h, hPTC-mCherry was trypsinized (Euroclone) and analyzed at FACS. All isotypes controls are shown in Supplemental Fig. S5.

### Human Monocyte Purification

Monocytes CD14+ were positively selected by magnetic cell sorting MACS (Miltenyi Biotec) from peripheral blood mononuclear cells (PBMNCs) derived from buffy coats of healthy donors, according to the manufacturing instructions. Purity of CD14+ cells was checked by flow cytometry staining (BD LSR II) and it was >95%. CD14+ cells were resuspended in RPMI (BioConcept) plus 5% FCS for coculture experiments.

### In Vitro Macrophage Differentiation

Monocytes CD14+ previously purified from PBMNC of healthy subjects were cultured in vitro to obtain macrophages. Furthermore, 3 × 10^6^ cells/well were cultured in RPMI plus 5% FCS in a six-well plate in the final volume of 6 mL/well in the presence of granulocyte-macrophage colony-stimulating factor (GM-CSF) of 10 ng/mL (7954-GM, R&D Systems) and placed in incubator at 37°C, 5% CO_2_. At *day 5*, culture supernatant was removed and adherent cells were recovered with cold PBS. Macrophage phenotype was checked by flow cytometry staining (BD LSR II) for scatter parameters and for the expression of CD14, CD16, CD80, CD84, CD64, and human leukocyte antigen–DR isotype (HLA-DR) (Supplemental Fig. S8). Macrophages were resuspended in RPMI plus 5% FCS for coculture experiments.

### Coculture Experiments

hPTC were seeded at a density of 25 × 10^3^ cells/24-well. The day after hPTC were stimulated with TGF-β1 for 24 h, or treated with hydrogen peroxide (0.5 µM, Merck) for 1 h to mimic hypoxia. After stimulation, the medium was removed, washed once with PBS, and fresh medium was added. Human primary fibroblasts were seeded at a density of 10^4^ cells on transwell permeable supports with pore sizes of 8 µm (Corning). After 24 h of incubation, cells were harvested, RNA was extracted, and analyzed by real-time PCR. For the monocyte and macrophage cocultures, hPTC were seeded at a density of 1 × 10^5^ cells/6-well. After stimulation as described earlier, 1 × 10^6^ monocytes or macrophages were seeded on transwell permeable supports with pore sizes of 3 µm (Corning).

### Quantitative Real-Time PCR

Total RNA from sorted cells and total mCherry-hPTC was extracted using RNeasy Microkit (Qiagen) and retrotranscribed using TaqMan Reverse Transcription Reagents (Thermo Fisher Scientific). TaqMan RT-PCR for 18S, TGF-β1, SMAD2, SMAD3, connective tissue growth factor (CTGF), VIMENTIN, musculoaponeurotic fibrosarcoma (MAF), hypoxia-inducible factor (HIF)1α, IL6, C-C motif chemokine ligand 2 (CCL2), and p21 was performed using customized TaqMan assays (Thermo Fisher Scientific) on a 7900HT Fast Real-Time (Applied Biosystem). ΔΔ*CT* was used to calculate relative quantification.

### Single-Cell Data Analysis

Two previously published datasets (GSE212273, GSE212275) containing single-cell data generated on mouse kidneys at 2 and 30 days after uni-IRI, and hPTC respectively, were reanalyzed to investigate the mechanisms linking polyploid TC to kidney fibrosis (4).

We started from the mice data set (GSE212273) that integrates samples from three experimental points *t0*, *t2*, and *t30*. To study events happening between *t2* and *t30*, by means of the Scanpy framework, we loaded the data set, removed the *t0* samples, and recalculated the neighborhood graph on the latent space to cluster and annotate the resulting data.

Next, we annotated the new data set, adding information obtained from the proximal TC analysis described in our previous work (4, 12). In particular, we looked for cells belonging to proximal *clusters 8* and *9*, as defined in the previous paper, and we labeled cells in the new data set as 8 or 9 based on the original proximal cluster. As a result, we identified polyploid cells in the new data set, that we used for the interactome analysis.

The human data set (GSE212275) was loaded in Scanpy. We focused our analysis on polyploid *clusters 4*, *5*, *7*, *9*, and *10* as previously demonstrated and described elsewhere (4). We further generated and analyzed a new data set containing hPTC stimulated with TGF-β1 or vehicle-treated cells (GSE242695). To start, raw sequencing data were processed using the 10× Genomics Cell Ranger pipeline (v.3.0.1). First, cellranger mkfastq demultiplexed libraries based on sample indices and converted the barcode and read data to FASTQ files. Second, cellranger count took FASTQ files and performed alignment to the human GRCh38 reference genome, to then proceed with filtering and unique molecular identifier (UMI) counting. Next, we loaded the count matrix in Scanpy to proceed with quality control. After filtering, we obtained 10,402 cells with less than 25% of mitochondrial read rate and expressing more than 2,000 genes. Cell-specific biases were normalized by dividing the measured counts by the size factor obtained through the scran computeSumFactors method, which implements the deconvolution strategy for scaling normalization (4). Finally, all counts were log-transformed after the addition of a pseudocount of 1. Next, we mitigated the batch effect through the matching mutual nearest neighbors (MNN) algorithm to later proceed with feature selection to keep “informative” genes only, used for dimensional reduction through PCA. The first 50 principal components (PCs) were used to construct a neighborhood graph of observations through the pp.neighbors function, which relies on the Uniform Manifold Approximation and Projection (UMAP) algorithm to estimate connectivity of data points. Cell cycle analysis was performed by creating two lists of genes associated to the S and G2/M phases based on cell cycle genes previously defined (16), passed to the tl.score_cell_cycle_genes function to score S and G2/M phases. Next, we clustered data by tl.louvain function at different resolutions (0.5, 1) and 1 proved to be the best, producing 10 clusters of hPTC. To annotate the clusters, we ran the tl.rank_gene_groups function using the Wilcoxon rank-sum method, to define the marker genes of each cluster. The same was conducted for the treated and untreated cells, to define the marker gene sets of each group and use them for a gene set enrichment analysis (GSEA), conducted through the prerank function implemented in the gseapy python library (17), that we used to query the MSigDB_Hallmark_2020 database.

### Mouse Interactome Analysis

The mouse datasets prepared before were used to conduct an interactome analysis with CellPhoneDB 4.0.0, through the command: cellphonedb method statistical analysis meta.tsv counts.tsv, where “meta.tsv” corresponds to the metadata, reporting the cell types that have been exported, and “counts.tsv” to the normalized read counts file. The outputs produced by CellPhoneDB for the *t2* and *t30* data, and stored in two “significant means.txt” files, were loaded as pandas data frames in a python environment (https://pandas.pydata.org) and analyzed separately.

The data frames report the interactions observed between each cell type (rows), at the two experimental points, respectively. The interactions are defined based on the coexpression of a “ligand” molecule in a cell type, defined source, and a “receptor” molecule in another one, defined target. To produce a matrix reporting all “as-source” and “as-target” interactions for each cell type, we extended both data frames by adding a source and a target column, which allowed us to obtain pivot tables reporting the number of interactions between “as-source” (rows) and “as-target” (columns) cell types. With these tables, we computed the Spearman’s Rank Correlation for rows and columns. Next, we used each data frame to obtain “as-source” and “as-target” interaction profiles for each cell type. To this aim, we merged the “as-source” (or “as-target”) cell types with the interaction IDs (CPI-SC03515B178), producing a matrix made by columns consisting of the specific interactions of each cell type (CPI-SC03515B178Proximal Tubule) and rows consisting in the cell types. This matrix allowed us to understand if an interaction observed in a specific cell type was also present in another or not, and consequently define specific cell type interaction profiles. Also in this case we computed the Spearman’s Rank Correlation for rows and columns.

Finally, we encoded the interaction sets as multipartite graphs, defined as a 3-tuple G = (C,M,E), where C are the cell types, M the molecules (ligand/receptors), and E the edges connecting the elements of the graph. To generate such a graph, we iterated over the rows of the data frame and added each interaction as a path, defined as a sequence of edges connecting a source cell (e.g., Proximal Tubule) to a ligand (e.g., FLT1), a ligand to a receptor (e.g., VEGFB), and a receptor molecule to a target cell (e.g., Endothelial).

CellPhoneDB classifies interactions as directed if one of the partners is a ligand and the other is a receptor and undirected otherwise. In this graph, we preserved this classification producing a single path (e.g., Proximal Tubule, FLT1, VEGFB, Endothelial) if directed and two paths, one reversed, if not. For each edge, a “weight” *W* was defined to encode the total number of occurrences of the corresponding interactions in the interactome. The two paths of an undirected interaction are counted only once for the purpose of computing *W*. The networks were assembled using the NetworkX library (18), and analyzed with the graph algorithms implemented in the library. To compute the centrality measures we used the degree_centrality, in_degree_centrality, and out_degree_centrality functions. The betweenness centrality was calculated through the betweenness_centrality function, using the inverse of the edge weights as a distance measure; the katz_centrality numpy function was used to compute the Katz centrality, with the edge weights as the weight measure, and the attenuation factor α as the reciprocal of the absolute value of the largest eigenvalue of the network adjacency matrix.

Finally, by comparing the *day 2* and *day 30* interactomes, we defined the absent, stable, lost, and gained interactions for each cell population. Processing this data as a network, we defined the top-changing reactions from *day 2* to *day 30*.

### Statistical Analysis

Comparison between groups was performed by the Mann–Whitney test or Student’s *t* test. A *P* value < 0.05 was considered statistically significant. Statistical analysis was performed using OriginPro (RRID:SCR_015636) statistical software.

## RESULTS

### Polyploid TC with DNA Damage Accumulate after Injury In Vivo

**Figure 1.**
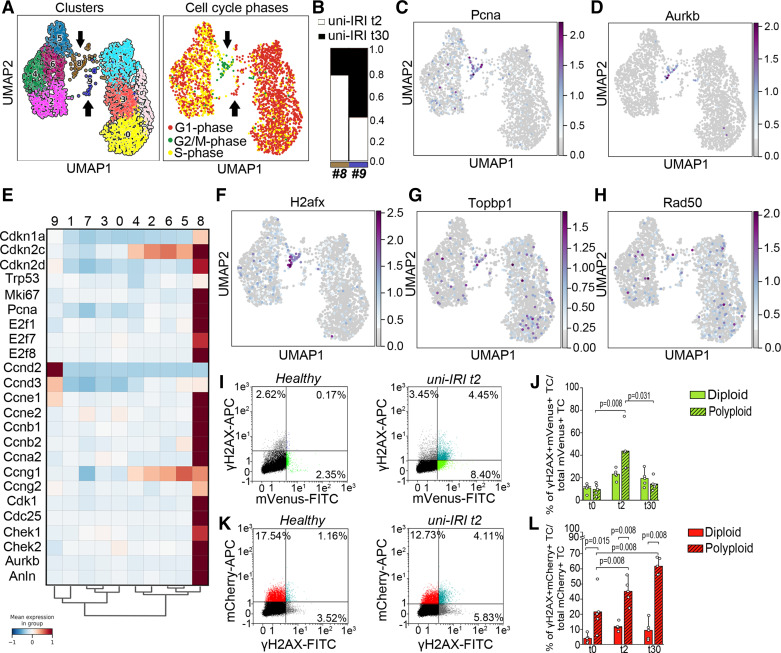
Polyploid tubular cells (TC) with DNA damage accumulate after injury in vivo. *A*: Uniform Manifold Approximation and Projection (UMAP) of cluster distribution and of cell cycle distribution of mouse proximal tubular cells (PTC) at *day 2* and *30* after unilateral ischemia reperfusion injury (uni-IRI). *B*: barplot showing experimental time distribution in *cluster 8* and *cluster 9*. UMAP distribution of cell cycle activation (Pcna) (*C*), and cell cycle progression (Aurkb) genes (*D*). *E*: matrixplot showing expression of genes involved in cell cycle progression and inhibition. *F*–*H*: UMAP distribution of DNA damage markers H2afx, Topbp1, and Rad50. *I*: representative FACS analysis and gating strategy of mVenus+ TC stained for γH2AX in healthy and 2 days after uni-IRI (*n* = 5). *J*: percentage of γH2AX+/mVenus+ TC diploid (i.e., actively proliferating) and polyploid (i.e., undergoing endoreplication). *K*: representative FACS analysis and gating strategy of mCherry+ TC stained for γH2AX in healthy and 2 days after uni-IRI (*n* = 5). *L*: percentage of γH2AX+/mCherry+ TC diploid and polyploid showing accumulation over time in the polyploid population. Statistical significance was calculated by two-sided Mann–Whitney test; numbers on graphs represent exact *P* values. Bar plots: line = mean, whisker = outlier (coef. 1.5). *n* = number of mice.

After AKI, a subset of polyploid TC undergoes continuous endoreplication cycles and becomes senescent and profibrotic over time (4). These TC can be identified by combining the detection of cell cycle phases using the FUCCI2aR technology with the quantification of the DNA content (4, 12) (Supplemental Figs. S1 and S2). To dissect the mechanisms linking cycling polyploid TC to kidney fibrosis, we reanalyzed the datasets generated on mouse kidneys at 2 (acute phase) and 30 days (chronic phase) after unilateral ischemic reperfusion injury (uni-IRI), restricting our analysis to proximal TC (PTC) and in particular to the clusters we have shown to be polyploid (GSE212273) (4) (Fig. 1*A*). Louvain clustering showed the presence of 10 clusters and *clusters 8* and *9* represented polyploid TC (4) (Fig. 1*A*). Interestingly, the analysis of cell cycle distribution showed that *cluster 9* was mostly composed by polyploid PTC at *day 30* in the G1 phase of the cell cycle (Fig. 1, *A* and *B*). Conversely, *cluster 8* was mostly composed of polyploid PTC at *day 2* (Fig. 1, *A* and *B*) that appeared to be actively cycling based on cell cycle scoring algorithm (16) (Fig. 1*A*) and on the expression of traditional cell cycle activation markers (Pcna) and G2/M phase markers (Aurkb) (Fig. 1, *C* and *D*). Polyploid *cluster 8* was further characterized by both polyploidy regulators (E2f1, E2f7, E2f8, Ccne1, Ccne2, and Cdk1) and cell cycle inhibitors such as p21 (Cdkn1a) and p19 (Cdkn2d) genes, proving that this cluster was not actively proliferating but rather undergoing polyploidization (Fig. 1*E*). This result demonstrates that traditional cell cycle markers identify a polyploid TC population at *day 2* after AKI. As p21 expression was shown to be induced in DNA-damaged cells (6), we analyzed the expression of a panel of DNA damage markers (Fig. 1, *F–H*). Among those, H2afx was also primarily restricted to *cluster 8* (Fig. 1*F*), demonstrating that DNA-damaged TC mostly undergo endoreplication-mediated polyploidization after AKI. To confirm this observation, we took advantage of our model of Pax8/FUCCI2aR mice, where polyploid TC can be identified as mCherry+ TC with ≥4C DNA content (polyploid TC in G1 phase) or mVenus+ with ≥8C DNA content (polyploid TC in G2/M phase), as previously described (4) and shown in Supplemental Fig. S2, *C* and *D*. Importantly, we found that the percentage of TC with DNA damage (TC positive for γH2AX) significantly increased among the polyploid TC in G2/M phase (mVenus+ with ≥8C DNA content) compared with proliferating diploid TC (mVenus+ with a DNA content = 4C) at *day 2* after uni-IRI, but decreased 30 days after uni-IRI (Fig. 1, *I* and *J* and Supplemental Fig. S3). This indicates that immediately after AKI, DNA damage accumulates in polyploid, but not in truly proliferating TC. Conversely, the percentage of G1 polyploid TC with DNA damage (TC positive for γH2AX) progressively increased 30 days after uni-IRI (mCherry+ TC with ≥4C DNA content) (Fig. 1, *K* and *L* and Supplemental Fig. S3), suggesting that after AKI polyploid TC with DNA damage progressively accumulate and rest in the G1 phase of the cell cycle. By contrast, no significant upregulation of γH2AX was found in diploid TC (mCherry+ with a DNA content = 2C) (Fig. 1*L*). Consistently, polyploid TC with a ≥8C DNA content has significantly increased at *day 30* after AKI, suggesting that polyploid TC with DNA damage undergo further endoreplication cycles to promote polyploidization overtime (Supplemental Fig. S2*D*). Taken altogether, these results demonstrate that *1*) expression of G2/M cell cycle markers in the acute phase of injury response characterizes endoreplicating rather than proliferating TC; *2*) polyploid TC but not diploid TC accumulates DNA damage in the acute phase after injury, progressively increasing over time, and finally stalls in the G1 phase of the cell cycle, suggesting that DNA damage stimulates TC to undergo endoreplication cycles becoming polyploid.

### Polyploid TC Are Profibrotic and Actively Produce TGF-β1 In Vitro

**Figure 2.**
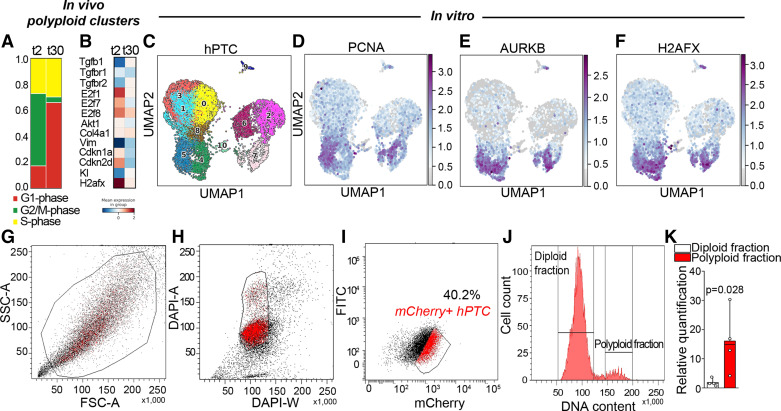
Polyploid tubular cells (TC) with DNA damage are profibrotic and actively produce transforming growth factor-β1 (TGF-β1) in vitro. *A*: barplot showing cell cycle phases in mouse polyploid clusters (8 and 9) divided in *day 2* (*t2*) and *30* (*t30*) after unilateral ischemia reperfusion injury (uni-IRI). *B*: matrixplot of mouse polyploid clusters (8 and 9) divided in *day 2* (*t2*) and *30* (*t30*) after uni-IRI, showing TGF-β1 and its receptors. *C*: Uniform Manifold Approximation and Projection (UMAP) showing human proximal tubular epithelial cell (hPTC) clusters. UMAP distribution of cell cycle activation (PCNA) (*D*), and cell cycle progression (AURKB) genes (*E*). *F*: UMAP distribution of DNA damage marker H2AFX. *G*–*I*: FACS analysis of mCherry-hPTC (cells in the G1 phase) showing the gating strategy for sorting. *J*: cell cycle distribution of diploid and polyploid mCherry-hPTC. A representative experiment out of 4 is shown. *K*: TGF-β1 gene expression in sorted polyploid mCherry-hPTC over diploid mCherry-hPTC (*n* = 4). Statistical significance was calculated by two-sided Mann–Whitney test; numbers on graph represent exact *P* value. Bar plots: line = mean, whisker = outlier (coef. 1.5). *n* = number of experiments.

To dissect the link between polyploid TC with DNA damage and fibrosis development, we analyzed the expression of genes known to be involved in the development of fibrosis in mouse kidneys at 2 and 30 days after uni-IRI. Importantly, G1 polyploid TC at *day 30* after uni-IRI appears to express TGF-β1 and its receptor (TGF-βR2, Fig. 2, *A* and *B*), indicative of an acquired profibrotic state. Conversely, diploid TC were not characterized by TGF-β1 expression (Supplemental Fig. S4*A*). Likewise, a reanalysis of the data set (GSE212273) of the human proximal tubular cell (hPTC), which we have found to contain a fraction of polyploid hPTC (4), showed similar results (Fig. 2, *C–F*). Unsupervised clustering of primary hPTC showed the presence of 11 clusters, and *clusters 4*, *5*, *7*, *10*, and *9* were identified as polyploid clusters based on the expression of characteristic genes involved in TC polyploidization (4, 19) (Fig. 2*C*). In agreement with what we had observed in vivo, polyploid clusters were characterized by cell cycle activation markers (Pcna, Aurkb), polyploidy regulators (E2f1, E2f7, E2f8, Ccnb1, Cdk1) (Fig. 2, *D* and *E* and Supplemental Fig. S4*B*) along with the DNA damage marker, H2afx, confirming that DNA damage accumulates preferentially in polyploid TC (Fig. 2*F*). This proves that in vitro polyploid TC can be successfully used to mimic the in vivo setting. To verify if polyploid TC actively produce TGF-β1, we transduced hPTC with a mCherry-G1 vector to identify cells in the G1 phase (Fig. 2, *G–J*), as previously described (4). Upon transduction, hPTC express the fluorescent protein mCherry (red) in the nuclei of cells in G1 (from now on indicated as mCherry-hPTC), allowing to discriminate G1-polyploid cells (mCherry-hPTC with ≥4C DNA content) from G1-diploid cells (mCherry-hPTC with 2C DNA content) (Fig. 2, *G–J* and Supplemental Fig. S5, *A*–*C*). Importantly, a marked upregulation of mRNA expression of fibrogenic growth factor encoding TGF-β1 was observed in sorted polyploid mCherry-hPTC compared with diploid mCherry-hPTC (Fig. 2*K*). Collectively, these data validate the results observed in vivo and demonstrate that G1 resting polyploid TC actively produced TGF-β1.

### Polyploidization Is a Self-Sustained Mechanism Stimulated by TGF-β1

**Figure 3.**
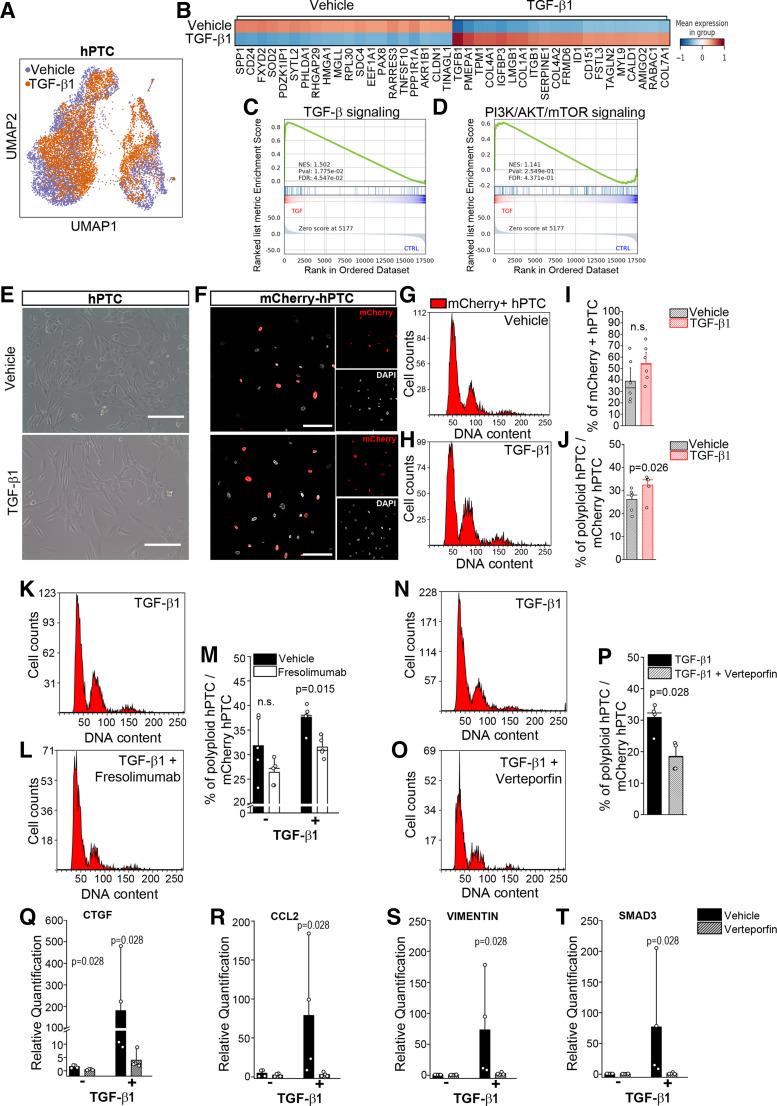
Polyploidization is a self-sustained mechanism stimulated by transforming growth factor-β1 (TGF-β1). *A*: Uniform Manifold Approximation and Projection (UMAP) showing sample distribution. *B*: first 20 characteristic genes of vehicle-treated human proximal tubular epithelial cell (hPTC) and TGF-β1-treated hPTC. *C*: gene set enrichment analysis showing activation of TGF-β pathway. *D*: gene set enrichment analysis showing activation of AKT pathway, one of the regulator of polyploidy in PTC. *E*: representative brightfield picture of mCherry-hPTC treated with vehicle (*top*) or TGF-β1 (*bottom*) for 48 h. Bar = 400 µm. *F:* representative picture of mCherry-hPTC treated with vehicle (*top*) or TGF-β1 (*bottom*) for 48 h. DAPI counterstains nuclei. Bar = 150 µm. *G*: cell cycle distribution of vehicle-treated mCherry-hPTC. *H*: cell cycle distribution of TGF-β1-treated mCherry-hPTC. *I*: total percentage of mCherry-hPTC in vehicle and TGF-β1 conditions (*n* = 6). *J*: percentage of polyploid mCherry-hPTC in vehicle-treated or TGF-β1-treated culture (*n* = 6). *K*: cell cycle distribution of TGF-β1-treated mCherry-hPTC. *L*: cell cycle distribution of TGF-β1 and Fresolimumab-treated mCherry-hPTC. *M*: total percentage of mCherry-hPTC in vehicle and TGF-β1 conditions (*n* = 5). *N*: cell cycle distribution of TGF-β1-treated mCherry-hPTC. *O*: cell cycle distribution of TGF-β1 and Verteporfin-treated mCherry-hPTC. *P*: total percentage of mCherry-hPTC in TGF-β1 and TGF-β1 with Verteporfin conditions (*n* = 4). *Q*–*T*: real-time PCR analysis of CTGF, CCL2, VIMENTIN, and SMAD3, following TGF-β1 stimulation and verteporfin treatment (*n* = 4). Statistical significance was calculated by two-sided Mann–Whitney test; numbers on graphs represent exact *P* values. Bar plots: line = mean, whisker = outlier (coef. 1.5). *n* = number of experiments; CTGF, connective tissue growth factor; CCL2, C-C motif chemokine ligand 2.

As polyploid TC with DNA damage progressively increase over time along with an increase in TGF-β1 expression, we investigated whether TGF-β1 directly promotes polyploidization. To this aim, we performed scRNA-Seq analysis on hPTC stimulated with TGF-β1 or vehicle. Following TGF-β1 stimulation, hPTC expressed TGF-β1 and its downstream targets as expected (Fig. 3, *A* and *B* and Supplemental Table S1). Moreover, TGF-β1-treated hPTC were characterized by a differential expression of profibrotic genes (Supplemental Table S1, light yellow genes), which we had recently showed to be characteristic of polyploid hPTC and were enriched with hypertrophy genes (Supplemental Table S1, light blue genes), indicative of polyploidization. Consistently, a gene set enrichment analysis, confirmed the activation of TGF-β and AKT pathways (Fig. 3, *C* and *D*), which we have shown to be a key player in TC polyploidization (4). To definitely prove that TGF-β1 stimulates the acquisition of polyploidization, we then treated mCherry-hPTC with TGF-β1 for 48 h and observed that the fraction of polyploid TC significantly increased compared with vehicle-treated hPTC (Fig. 3, *E–J* and Supplemental Fig. S5, *D*–*I*,) proving that TGF-β1 stimulates hPTC polyploidization. Consistently, treatment of mCherry-hPTC with Fresolimumab, a TGF-β1 neutralizing antibody, significantly reduced the percentage of polyploid TC after TGF-β1 stimulation (Fig. 3, *K*–*M* and Supplemental Fig. S6). Treatment with verteporfin, a YAP1 inhibitor, was sufficient to reduce TGF-β1-stimulated polyploidization, implying that TGF-β1 promotes polyploidy via YAP1 (Fig. 3, *N*–*P*). In addition, treatment with verteporfin prevented the upregulation of profibrotic genes following TGF-β1 stimulation (Fig. 3, *Q*–*T*). Collectively, these data proved that TC polyploidization is a self-sustained mechanism mediated by TGF-β1 via YAP1 activation.

### Polyploid TC Interact with Macrophages and Fibroblasts to Sustain Tubulointerstitial Fibrosis

**Figure 4.**
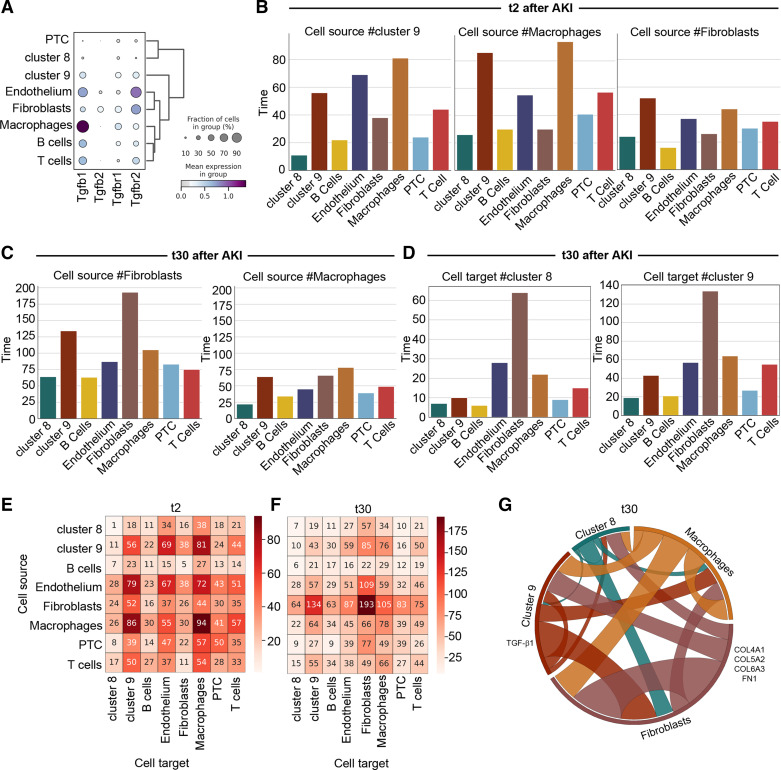
Polyploid tubular cells (TC) interact with macrophages and fibroblasts to sustain tubulointerstitial fibrosis. *A*: dotplot showing transforming growth factor (Tgf)-β1, Tgf-β2, Tgf-βr1, and Tgf-βr2 distribution in all the mouse populations retrieved from kidneys 2 and 30 days after unilateral ischemia reperfusion injury (uni-IRI). *B*: barplots reporting the number of interactions occurring 2 days after uni-IRI between *cluster 9*, macrophages, and fibroblasts as source and all the cell types. *C*: barplots reporting the number of interactions occurring 30 days after uni-IRI between fibroblasts and macrophages as source and all the cell types. *D*: barplots reporting the number of interactions occurring 30 days after uni-IRI between the *cluster 8* and *9* cells as target, and all the cell types. *E* and *F*: heatmap reporting the number of interactions between cell types, as source and target, at *day 2* and *30* after uni-IRI. *G*: Circos plot of ligand-receptor interactions among polyploid *cluster 9*, *8*, macrophages, and fibroblasts in kidneys at *day 30* after uni-IRI. The populations producing the putative ligand (TGFB1-AR, COL4A1-Integrin a1b1, COL5A2-Integrin a1b1, COL6A3-Integrin a1b1, and FN1-Integrin aVb1) are shown. PTC, proximal tubular cells.

**Figure 5.**
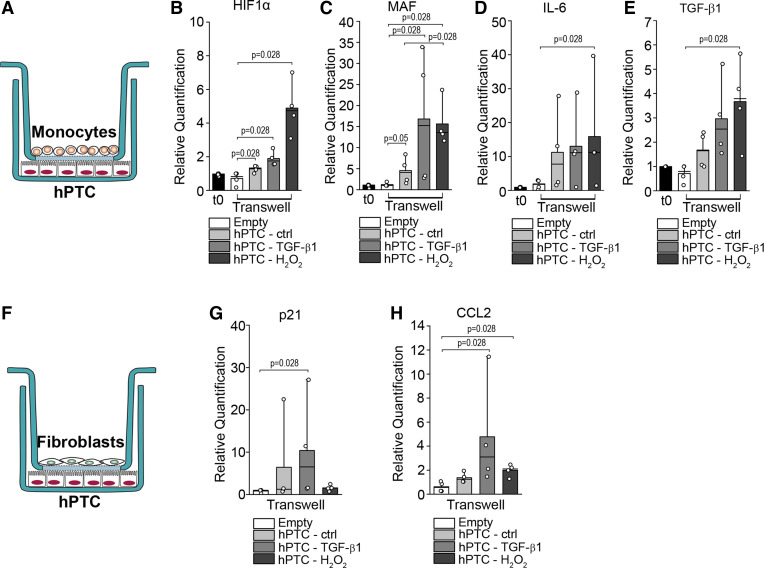
Tubular cells (TC) interact with monocytes and fibroblasts activating proinflammatory and profibrotic pathways. *A*: schematic representation of experimental plan for coculture with monocytes. Real-time PCR analysis of hypoxia-inducible factor (HIF)1α (*B*), MAF(*C*), IL-6 (*D*), and transforming growth factor (TGF)-β1 (*E*) in monocytes following coculture with nonstimulated human proximal tubular epithelial cells (hPTC) and after TGF-β1 and H_2_O_2_ treatment (*n* = 4). *F*: schematic representation of experimental plan for coculture with fibroblasts. *G* and *H*: real-time PCR analysis of p21 and CCL2 in fibroblasts following coculture with nonstimulated hPTC and after TGF-β1 and H_2_O_2_ treatment (*n* = 4). Statistical significance was calculated by two-sided Mann–Whitney test; numbers on graphs represent exact *P* values. Bar plots: line = mean, whisker = outlier (coef. 1.5). Empty: transwell with monocytes or fibroblasts without hPTC; *t0*: monocytes after purification. *n* = number of experiments; MAF, musculoaponeurotic fibrosarcoma.

**Figure 6.**
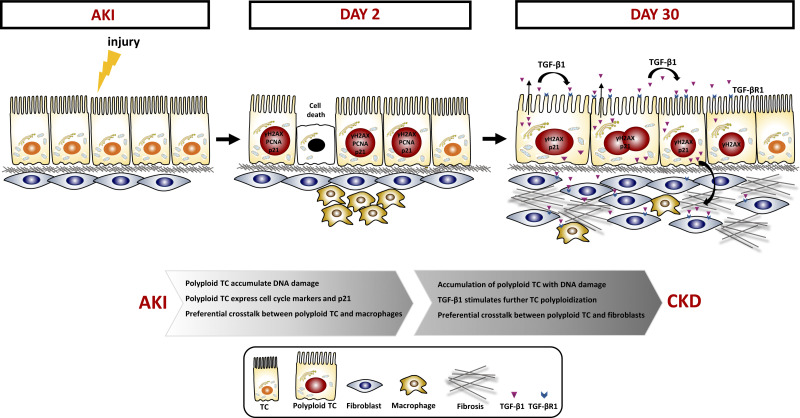
Schematic representation of polyploid tubular cells (TC) response to acute kidney injury (AKI). In response to AKI, TC undergo polyploidization. Polyploid cells are characterized by DNA damage, cell cycle markers and p21 expression 2 days after AKI. In the long-term, polyploid TC start to secrete transforming growth factor (TGF)-β1, which triggers a feedback loop to generate further polyploid TC and activate macrophages and fibroblasts. CKD, chronic kidney disease; γH2AX, H2A Histone family member X; p21, CDKN1A; TGF-βR1, transforming growth factor β receptor 1.

As TGF-β1 is known to stimulate fibroblast and inflammatory infiltrate activation, we leveraged our data set generated on mouse kidneys at 2 and 30 days after uni-IRI, to explore the interaction between polyploid TC, fibroblasts, and proinflammatory populations to promote fibrosis. To do so, we included in our scRNA-Seq analysis inflammatory cells, fibroblasts, and endothelial cells (Supplemental Fig. S7, *A* and *B*) and checked the expression of Tgf-β1 and its receptors. Polyploid *cluster 9* and all interstitial cells produced Tgf-β1, Tgf-βr1, and Tgf-βr2, with macrophages and endothelial cells being the major producers, suggesting a cross talk among these populations (Fig. 4*A*). Conversely, the diploid PTC did not express Tgf-β1 (Fig. 4*A*). To quantify cell-cell communication networks, we then performed ligand-receptor analysis with CellphoneDB. On *day 2* after AKI, a total of 2,230 interactions took place between all the pairwise combinations of the cell types (Fig. 4*B*). Interestingly, we found that macrophages are the main target of polyploid *cluster 9* and vice versa, suggesting an active contribution of polyploid TC in the recruitment of macrophages (Fig. 4*B*). Moreover, whereas fibroblasts interacted with all populations, polyploid *cluster 9* appeared to be a fibroblast preferential target (Fig. 4*B*). Conversely, polyploid *cluster 8* appeared to interact weakly with macrophages, endothelial cells, and fibroblasts (Supplemental Fig. S7*C*). To understand the extent of the perturbations of such network, we performed the same analysis on *day 30*. As expected, interactions of fibroblasts greatly increased with all the populations (67%), whereas macrophage interactions decreases by 5%, suggesting a progressive shift in the injury response (Fig. 4*C*). Importantly, polyploid *clusters 8* and *9* were exclusive targets of fibroblasts (Fig. 4*D*, Supplemental Fig. S7*D*). Remarkably, the interactions of polyploid *cluster 9* increased by 61% specifically only with fibroblasts from *day 2* to *day 30* (Fig. 4, *E–G* and Supplemental Fig. S7*E*), supporting the existence of a cross talk between fibroblasts and polyploid TC that progressively grows after AKI. Moreover, a detailed analysis of the top changing reactions between the polyploid *cluster 9* and fibroblasts confirmed a dominant role of Tgf-β1 signaling (TGFB1-AR) and profibrotic pathways (COL4A1-Integrin a1b1, COL5A2- Integrin a1b1, COL6A3- Integrin a1b1, and FN1-Integrin aVb1) (Fig. 4*G* and Supplemental Fig. S7*F*). The same analysis performed between *day 2* and *day 30* in polyploid *cluster 9* confirmed a progressive shift in the injury response, featuring the increase of profibrotic pathways along with the attenuation of proinflammatory pathways at *day 30* after AKI (Supplemental Fig. S7*G*). Collectively, these findings reveal the existence of a cross talk between polyploid TC, macrophages, and fibroblasts and indicate that polyploid *cluster 9* progressively modifies its state, passing from a proinflammatory profile at *day 2* recruiting macrophages to a profibrotic profile at *day 30* after AKI, activating fibroblasts. Of note, the observation that polyploid TC are progressively specific targets of fibroblasts, suggests a role for these cells in stimulating the continuous recruitment of polyploid TC over time and the acquisition of a fibrogenic phenotype, driving CKD. To corroborate the in vivo observation, we set up cocultures between hPTC and human monocytes or fibroblasts in vitro. Specifically, we treated hPTC with hydrogen peroxide to mimic the hypoxic injury or TGF-β1, and then we cocoltured them with human monocytes or fibroblasts, respectively. After coculturing with hPTC, monocytes acquired a proinflammatory phenotype, as demonstrated by upregulation of HIF1α, MAF, IL-6, and TGF-β1 compared with monocytes cultured alone (Fig. 5, *A–E*), and fibroblasts expressed higher levels of the senescent marker p21, and the profibrotic CCL2, compared with fibroblasts cultured alone (Fig. 5, *F–H*). Similar data were obtained with hPTC and macrophages cocultures, further proving that hPTC interact with macrophages activating profibrotic pathways (Supplemental Fig. S8). Collectively, these results confirmed the existence of a cross talk between polyploid TC and interstitial cells (Fig. 6).

## DISCUSSION

Kidney tubule response to AKI is still a matter of debate and has attracted a growing interest in recent years. Recognition that the tubule is not able to fully regenerate after AKI and that AKI itself is a risk factor for CKD stimulated the identification of previously unknown mechanisms of response within the tubule. Accordingly, our group recently proved the presence of polyploidy in TC after AKI and proposed them as a primary driver of CKD progression after AKI (4, 12). However, the mechanism linking polyploid TC to fibrosis development, the final manifestation of CKD progression, remains to be clarified. This work extends our previous studies on TC polyploidization and provides novel mechanistic insights, drawing out two main conclusions. First, expression of cell cycle markers identifies a population of DNA-damaged polyploid TC rather than proliferating TC. Indeed, using scRNA-Seq we showed that PTC that appeared to be actively cycling based on traditional cell cycle markers were rather polyploid TC with DNA damage. These cells were characterized by both polyploidy regulators and cell cycle inhibitors such as p21, which were found to promote TC polyploidization in a model of karyomegalic interstitial nephritis (6). In this model, TC are unable to successfully repair DNA damage promoting CKD (6). As in the heart and in the liver, the archetypes of polyploid organs, DNA damage in TC likely promotes polyploidization to endure oxidative stress (20, 21). Accordingly, polyploid TC tend to accumulate genome instability and survive after AKI, whereas diploid TC do not. Therefore, polyploidization may signal the presence of DNA damage, offering an opportunity for novel therapies, also in the kidney.

Second, we showed that polyploid TC with DNA damage acquire a progressive profibrotic profile characterized by TGF-β1 expression during the chronic phase after injury. Accordingly, DNA damage triggers TGF-β1 production initiating a vicious circle that leads to fibroblast activation and fibrosis development in the intestine (11). Importantly, we demonstrated that TGF-β1 directly promotes polyploidization via YAP1, thus suggesting TC polyploidization is a self-sustained mechanism promoted by TGF-β1. A similar mechanism was shown in the liver (9). The ligand-receptor analysis further revealed that TGF-β1 signaling fosters a reciprocal activation loop among polyploid TC, macrophages, and fibroblasts. It is reasonable to hypothesize that TGF-β1 secreted by polyploid TC and interstitial cells may be acting in an autocrine and paracrine fashion to the surrounding cells. Specifically, as diploid TC are also a target of TGF-β signaling, TGF-β1 can act to continuously increase the fraction of polyploid TC after AKI. This results in the activation of fibroblasts, which in turn interact with polyploid TC to maintain this loop. The existence of a preferential cross talk among polyploid TC, macrophages, and fibroblasts confirms a role for polyploid TC in promoting and accelerating the development of tubulointerstitial fibrosis in CKD. These results contribute to the ongoing revision of the paradigm of kidney tubule response to AKI. Previous studies concluded that after AKI, the mechanism driving CKD was the G2/M cell cycle arrest of TC in response to damage. However, we and others recently argued against this hypothesis (4, 12, 22), disproving a G2/M-arrested state of TC after injury and suggesting that arrested TC rather represents polyploid TC (4, 6, 12). Collectively, previous studies in conjunction with the results reported in this, suggest that G2/M-arrested cells and polyploid TC are the same rather than distinct TC states. Therefore, polyploid TC represent the apical determinant for the long-term outcome of kidney insults further reinforcing the central role played by kidney tubule in CKD and can represent a valid therapeutic target to slow its progression.

## DATA AVAILABILITY

Data used is available from the corresponding author upon reasonable request. Processed data for the human scRNA-Seq libraries generated in this study have been deposited in the Gene Expression Omnibus (GEO) database under accession code (GSE212273, GSE212275, GSE242695).

## SUPPLEMENTAL DATA

10.6084/m9.figshare.22200187 Supplemental Figs. S1–S8 and Supplemental Table S1: https://doi.org/10.6084/m9.figshare.22200187.

## GRANTS

This research was funded by the European Union’s Marie Sklodowska-Curie fellowship program under Grant Agreement No. 845774 (to L.D.C.). This study was also funded by PRIN: progetti di ricerca di interesse nazionale 2017T95E9X (to P.R.). L.D.C. is the recipient of L’Oreal-UNESCO for Women in Science award.

## DISCLOSURES

No conflicts of interest, financial or otherwise, are declared by the authors.

## AUTHOR CONTRIBUTIONS

L.D.C., P.R., and E.L. conceived and designed research; L.D.C., B.M., S.L., A.M., G.A., M.L.A., M.E.M., L.M., C.C., A.J.P., and V.R. performed experiments; L.D.C., R.S., B.M., S.L., A.M., G.A., M.E.M., L.M., L.C., A.M., F.A., P.R., and E.L. analyzed data; L.D.C., R.S., M.L.A., P.R., and E.L. interpreted results of experiments; L.D.C., R.S., and E.L. prepared figures; L.D.C., P.R., and E.L. drafted manuscript; L.D.C., R.S., P.R., and E.L. edited and revised manuscript; L.D.C., R.S., B.M., S.L., A.M., G.A., M.L.A., M.E.M., L.M., C.C., A.J.P., L.C., V.R., A.M., F.A., P.R., and E.L. approved final version of manuscript.
